# Large Scale Shrub Biomass Estimates for Multiple Purposes

**DOI:** 10.3390/life10040033

**Published:** 2020-03-30

**Authors:** Teresa Enes, José Lousada, Teresa Fonseca, Hélder Viana, Ana Calvão, José Aranha

**Affiliations:** 1 Centre for the Research and Technology of Agro-Environmental and Biological Sciences (CITAB), University of Trás-os-Montes and Alto Douro, 5001-801 Vila Real, Portugal; jlousada@utad.pt (J.L.); hviana@esav.ipv.pt (H.V.); j_aranha@utad.pt (J.A.); 2 Department of Forestry Sciences and Landscape Architecture (CIFAP), University of Trás-os-Montes and Alto Douro, 5001-801 Vila Real, Portugal; tfonseca@utad.pt; 3 Forest Research Centre (CEF), Instituto Superior de Agronomia, Universidade de Lisboa, Tapada da Ajuda, 1349-017 Lisboa, Portugal; 4 Department of Forestry Sciences, Agrarian Superior School, Polytechnic Institute of Viseu, 3500-606 Viseu, Portugal; 5 Águeda School of Technology and Management, University of Aveiro (ESTGA-UA), 3754–909 Águeda, Portugal; arc@ua.pt

**Keywords:** wildfires, productivity, bioenergy, allometric equations, *Pinus pinaster*

## Abstract

With the increase of forest fires in Portugal in recent decades, a significant part of woodlands is being converted into shrubland areas. *Background*: From an ecological point of view, woodlands and shrublands play an essential role, as they not only prevent soil erosion and desertification, but also contribute to soil protection, habitat preservation and restoration, and also increased biodiversity for carbon sequestration. Concerning the shrublands, the assessment of their biomass is essential for evaluating the fuel load and forest fire behavior and also beneficial for obtaining estimates of carbon and biomass for energy use. *Methods*: In this study, we collected data about the potential shrub biomass accumulation along fifteen years in former burnt areas within North Portugal. *Results*: The achieved results showed that for a post-fire period ranging from one to 15 years, the accumulated shrubs’ biomass ranged from 0.12 up to 28.88 Mg ha^−1^. The model developed to estimate the shrub biomass using the time after a fire (age) as a predictor variable presented a high adjustment to data (*p*-value of the F statistic <0.01 and R^2^ = 0.89), allowing estimating shrub biomass regeneration within former burnt areas with an RMSE of 3.31 Mg ha^−1^. *Conclusions*: This paper provides practical information on the availability and assessment of shrub biomass in North Portugal, highlighting the suitability of shrubs as potential sources of biomass.

## 1. Introduction

Due to its geographical location and morphological characteristics, mainly of hilly areas, Portugal has a large forest cover area of about 3.4 million hectares [1], with strong suitability for forest biomass production. Economic and socio-cultural factors, as well as an increasing migration of the rural population to large cities on the coast or to the main cities of the interior lead to the abandonment of agriculture and of forest areas and to the subsequent decline in land planning and management activities. This land abandonment led to gradual decreases in landscape diversity and complexity [2] and promoted biomass accumulation, which, in turn, increased the vulnerability to forest fires occurring during the dry seasons, typically from May to October [3,4]. In a country already prone to forest fire occurrence, due to the Mediterranean climate’s influence, the wildfires had a significant impact on short- and long-term land cover and use. According to the results of the Portuguese National Forest Inventory (NFI6) [5], in a 15 year time period, from 1995 to 2010, there was a net decrease in *Pinus pinaster* forest areas of 263,000 ha (26.6% of the original area), of which 85% was due to its conversion to the “shrublands and pastures” class of use.

The increase in wildfire severity in Portugal, reported in recent decades, raises concerns not only about the long-term adverse effects of fire recurrence in the environment [6,7], but it has also been demanding attention to this class of land cover. Shrubland areas currently cover more than two million hectares of the inland Portuguese territory [5]. Additionally, these new shrubland areas constitute the primary fuel for the frequent wildfires that take place in Portugal.

On the other hand, shrubs are essential drivers of forest ecosystem productivity and diversity. Forest understory vegetation is ecologically vital because shrubs, lichens, and mosses can have a direct effect on belowground processes such as decomposition, nutrient flow, and the accumulation of soil nutrients [8]. From an environmental point of view, shrublands play an important role, as they prevent soil erosion and desertification [9], as well as reduce soil erosion more effectively than trees [10]. They also contribute to soil protection [11], habitat preservation and restoration [12], increase biodiversity [13], and have a net positive effect on the recruitment of other species [12]. In the long term, shrub vegetation influences the fluxes of water and sediments by increasing the soil aggregate stability and cohesion and by improving water infiltration [14].

Post-fire shrub biomass regrowth evaluation may be, therefore, beneficial for the assessment of forest services and for calculating the estimates of carbon sequestration and potential energy stock, while also providing information about the shrub load fuel available, which might be useful for the prediction of fire behavior [15,16,17,18]. The regular harvesting of shrublands might, therefore, additionally be valuable for diminishing the substantial greenhouse gas emissions associated with these frequent wildfires [19] through the reduction of forest fire occurrence [20]. Moreover, the increasing interest in the use of forest biomass for bioenergy production in European countries, as an integrated strategy for mitigating climate change, increasing renewable energy security, and preventing forest fires, leads to a reinforced interest in the biomass of the shrubland areas.

The majority of forestry studies concerning biomass assessment are derived by means of allometric equations’ calculation, which focus solely on the trees’ biomass estimation. As stated by [21], although the tree biomass is the principle sink of carbon sequestration in forests, it is also necessary to account for shrub biomass, as these woody plants play an active role in ecosystem productivity.

A literature review about forest trees biomass and shrub biomass estimation enabled collecting several allometric equations (Table 1), for Portuguese regions [22,23], as well as for other countries (e.g., Central Greece [24] or Southern Spain [25]), were shrubs have different growth rates. The works in [22] to [25] studied and described the biomass accumulation for the main species occurring in these areas (e.g., species like *Cistus*, *Erica*, *Genista*, and *Ulex*; see Table 1). One of these previously presented equations [23] has the particularity of being able to estimate regenerated shrub biomass of former burnt areas up to 10 years post-fire. The authors surveyed 45 former burnt areas and adjusted an allometric equation (see Equation (10)), which can be used in association with a Geographical Information System project and estimate accumulated biomass in mapped burnt areas. Given that, in the last 30 years of forest fires in Portugal, fire recurrence ranged from 10 to 15 years [26,27,28,29,30]. The prediction of periods greater than 10 years is still required.

The main aim of the authors with the present paper was twofold. First, the study aimed to deepen the research about biomass assessment for shrubland cover class, supported by different databases in a Geographic Information System (GIS) project. Secondly, the authors aimed to develop a biomass model, which could extend the biomass predictions of shrubland cover using GIS, up to the age of 15 years, to embrace the maximum fire recurrence ranges registered in the country.

## 2. Materials and Methods

### 2.1. Study area Characteristics

**Figure 1:**
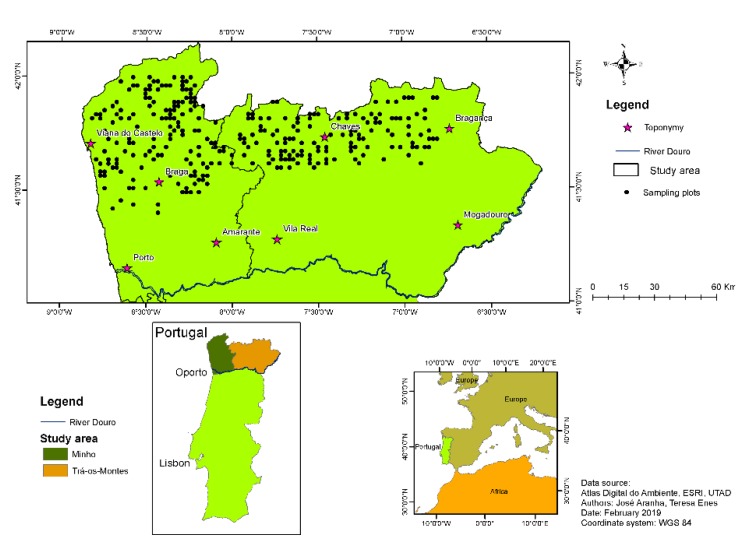
Distribution of sampling plots in the study area (North Portugal) and Portugal’s world geographical location.

The study area was comprised of 1.9 million ha (Figure 1) located in North Portugal. Although it was a relatively small region, there were edaphoclimatic and morphological asymmetries (Minho and Trás-os-Montes regions), as the altitude ranged from sea level (0 m) up to 1410 m in some mountains. With regard to soils, the origin, type, and characteristics varied greatly depending on the geographical area. The predominant soil types were Cambisols of both eruptive rocks and shales.

In flat areas crossed by rivers and streams, Fluvisol and Histosol areas were frequent, these areas being used for agricultural proposes.

In mountainous areas, the predominant soil types were: Rankers, Lithosols, and Luvisols. In some former burnt areas, where vegetation was reduced to scattered shrubs, bed rock was visible.

The Minho region is located in the NW of Portugal and borders Galicia (Spain) in the north and the Atlantic Ocean. This region is characterized as having an Atlantic wet climate, with relatively high exposure to Atlantic Ocean winds, high annual precipitation (1000–2400 mm), and mild summers (summer mean temperatures from 18 to 22 °C) [31].

Trás-os-Montes is located in the northeastern corner of the country. This region is characterized as having sub-continental prevailing climate conditions with long cold winters and short, but very hot and dry summers. The average annual rainfall ranges from 600 up to 1200 mm, and the annual mean air temperature is 10–14 °C.

### 2.2. Data Sources

This study was supported by 161 sampling plots installed in former burnt areas, within North Portugal, in the Minho and Trás-os-Montes regions (Figure 1), for shrub biomass classification and quantification.

In the first stage, a Geographic Information System (GIS) project was created using ArcGIS for Desktop (ESRI), in order to map former burnt areas from 1990 up to 2015, made available by the Institute of Nature Conservation and Forest (ICNF) and also by the European Forest Fire Information System (EFFIS). Then, all burnt areas polygons were isolated by burnt time (e.g., 1990, 1991, …, 2017) and submitted to a union routine in order to create a new topology for burnt areas’ combination and to enable fire recurrence calculation and post-fire time calculation.

In the second stage, this information was cross-referenced to CORINE Land Cover maps for 1990, 2006, and 2012 (CLC1990, CLC2006, and CLC2012, Copernicus 2018) and Portuguese Land Cover Maps COS 2010 in order to select only burnt areas where pre-fire land cover was shrublands or degraded forest lands.

The third stage was dedicated to a dynamic table creation that enabled classifying burnt areas previously selected according to fire recurrence, last fire occurrence date, and time spent after the fire. Then, 10 former burnt areas were selected per each year over the last fire occurrence.

The list of the steps is as follows:

1. Create a GIS project to manage burnt areas in inland Portugal.

2. Download burnt area limits shapefiles from 1990 up to 2016.

3. Create an independent shapefile for each year.

4. Perform a union routine in order to calculate fire recurrence and the year of the last occurrence.

5. Calculate the time elapsed since the last occurrence.

6. Identify 10 burnt scars per year after the last occurrence and for the past 15 years, throughout North Portugal.

7. Intersect the burnt areas’ shapefiles to land cover shapefiles in order to isolate only burnt patches in shrub areas.

8. Calculate the shapefiles’ centroid point in order to create a sampling network to support field work for biophysical data collection.

9. Perform field work for biophysical data collection by means of two perpendicular profile installations across each 500 m^2^ circular plot installed in each sampling plot.

10. Measure the 3 dimensions related to shrubs within the sampling plot: length, width, and height along each of the two perpendicular profiles. These values enable estimating shrub density within the sampling plot.

11. Cut the bush and shrubs at the base and weigh them with a field scale.

12. Collect shrub samples (trunk, branches, and leaves) to bring to the laboratory.

13. Dry the samples, in an open space until 30% moisture and weigh them with a laboratory scale.

14. Create an Excel table using the variables: time elapsed since the last fire occurrence, shrub density (percentage of land cover estimated during field work), wet weight, and dry weight.

15. Adjust the regression models in order to create an allometric equation suitable to be used for shrub weight estimation according to the time elapsed since the last fire occurrence.

**Figure 2:**
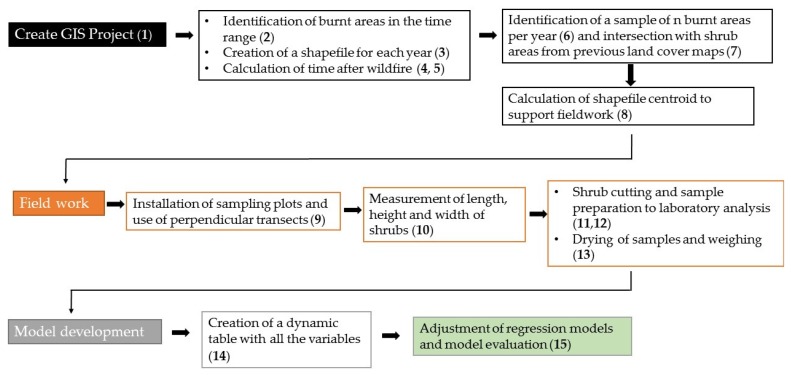
Graphic summary of the methodological steps followed in the study.

Figure 2 shows the diagram of the steps described.

### 2.3. Sampling Plots

In the Minho region, a total of 102 sampling plots were established and surveyed, while in the region of Trás-os-Montes, 59 plots were established and surveyed, based on former burnt areas’ location. Only plots with recurrence greater than or equal to 2 were visited to minimize the possibility of the natural regeneration of trees (e.g., *Pinus pinaster*, *Eucalyptus globulus*).

The temporary plots were of a circular shape with an area of 500 m^2^. In each plot, two perpendicular lines (transects) crossing the center were drawn, for shrub canopy density estimation and biometric measurements, such as shrub length, width, and height. Then, a selection of plants was cut off and weighed in the field in order to obtain the green weight of the sampled species (*Erica* sp., *Cytisus* sp., *Chamaespartium tridentatum*, and *Ulex* sp.) and to determine the biomass.

After the field measurements were completed, samples of each shrub species were stored in plastic bags, brought to the Forestry Sciences and Landscape Architecture of University of Trás-os-Montes and Alto Douro (UTAD) laboratory for further measurements. These samples were then placed in an oven at 70 °C in order to be dried out to 30% moisture. The results achieved, from field and laboratory measurements, were then used to update the GIS project database.

From this entire database, 150 plots (ten per year elapsed after fire occurrence) were selected in order to be submitted to regression analysis and allometric equations’ adjustment for biomass calculation. The potential set of regressors referred to the variable time elapsed after the fire (age) and, additionally, to the variables slope, aspect, and altitude, easily obtained from a Digital Elevation Model (DEM).

## 3. Results

### 3.1. Shrubland Characterization

**Figure 3:**
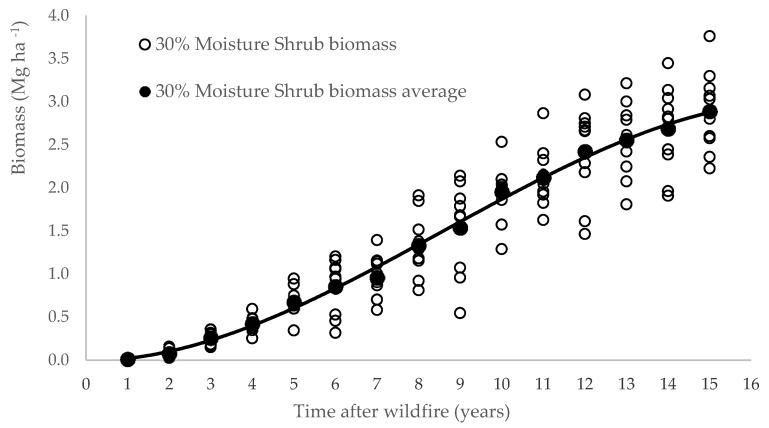
Accumulated biomass evolution over 15 years after fire and adjustment of the accumulated average amounts to Equation (16).

Results (Table 2 and Figure 3) showed that after one year post-fire, the soil was rarely covered by vegetation and the average biomass weight was around 0.12 Mg ha^−1^. Five years after fire occurrence, the ecosystem was able to regenerate a green canopy that covered near 80% of the land, and after 15 years of a forest fire, the shrubs’ canopy cover could reach 100% and accumulate around 28.9 Mg ha^−1^ (30% moister) of vegetal biomass.

### 3.2. Allometric Model for Shrub Biomass Estimation

In the first stage, post-fire age (t) and shrub biomass (B) variables were depicted in a bivariate plot to analyze the relationship type that could be established between age and biomass. The graphic representation showed a sigmoidal distribution, which led to the use of a non-linear approach.

In the second stage, several linear and non-linear regression models were adjusted by means of the Method of Least Squares (MLS) using JMP software and ranked according to the higher adjusted determination coefficient ($R_{adj}^{2}$) and lower Root Mean Squared Error (RMSE). From the potential set of regressors, “time post-fire” was retained as a significant explanatory variable. A third-degree polynomial model ($Y=\beta_{0}+\beta_{1}X+\beta_{2}X^{2}+\beta_{3}X^{3}+\varepsilon$) was selected as a proper model for the description of the relationship between B and t:

(16) $$B=-0.0461-0.0398t+0.3122t^{2}-0.0121t^{3}$$

$R_{adj}^{2}=0.8978$, $RMSE=3.31{\;\mathrm{Mg}\;\mathrm{ha}}^{-1}$, n=150 observations, *p*-value < 0.01.

where B is the shrub biomass (Mg ha^−1^ at 30% moister) and t is the time post-fire (years).

In the case of a polynomial model, the presence of multicollinearity was expected, and the statistical analysis carried out through the factors of inflation of the variance confirmed the existence of this. However, this phenomenon could only represent a problem when the model is applied to new data different from those that were used in the estimation of the model, which was not the case, due to the convenience of using regional models [32,33]. It should be advisable, therefore, to evaluate its performance (and proceed with adjustment of the values of the estimated parameters, if needed), before considering the use of the proposed model (Equation (16)) in different regions.

## 4. Discussion

### 4.1. Shrubland Characterization

In general, shrub canopy cover percentage varied according to shrub species present. If *Cytisus* sp. was the dominant species, five years after the fire, they reached 100% occupancy. In high mountain areas, where the dominant shrubs were mainly *Erica* sp. and *Chamaespartium tridentatum*, the coverage percentage hardly reached 100% (above an 800 m altitude, coverage rarely reached 100%). Altitude seemed to be a leading variable that controlled shrub canopy land cover. The higher the land, the lower the canopy density.

### 4.2. Allometric Model for Shrub Biomass Estimation

**Figure 4:**
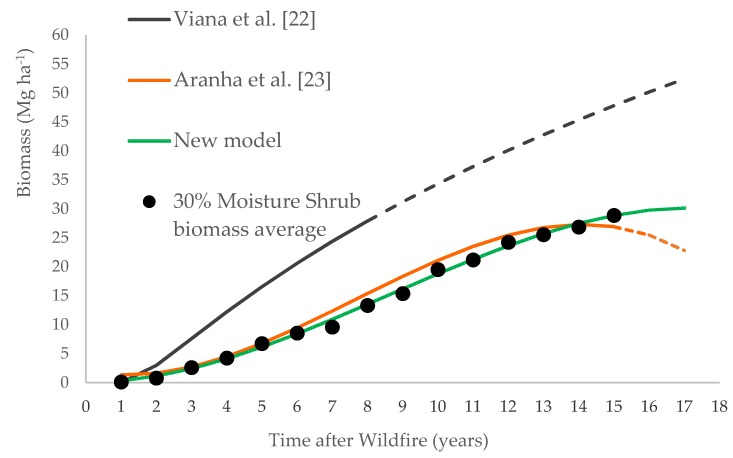
Graphic representation of biomass accumulated after wildfire during 17 years according to [22,23], Equations (8) and (10), respectively, and the new model, Equation (16). The black circles are the average values of shrub biomass (30% of moisture).

This research was driven by the need to adjust a new allometric equation to estimate regenerated shrub biomass since previously presented models (Viana et al. [22], Equation (8), and Aranha et al. [23], Equation (10)) were not accurate to be applied to the entire study area or for extended time periods. The new calculated model was compared to the previous ones in order to demonstrate its fitting adequacy, as presented in Figure 4.

**Figure 5:**
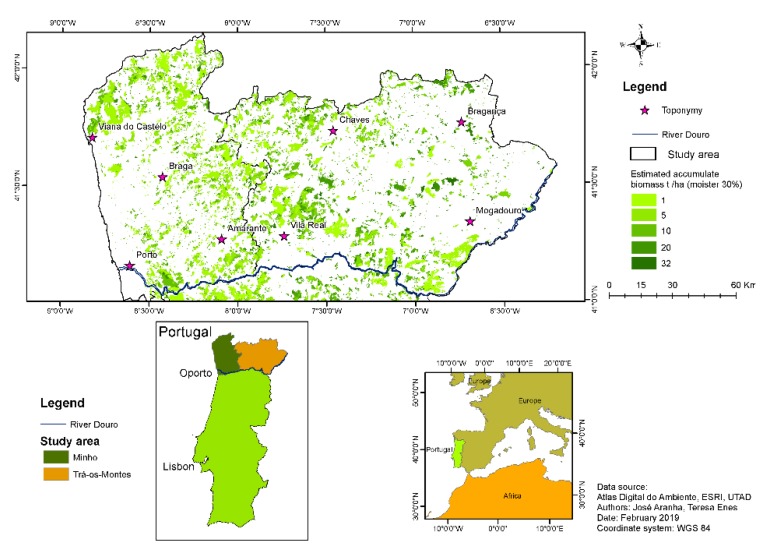
Spatial distribution of the amount of biomass accumulated in burned areas in the north of Portugal, during 27 years (1990–2017), expressed in Mg ha^−1^.

The equation adjusted by [22] was based on information collected from 18 sampling plots for age ranging from two to seven years. Thus, the estimates of biomass values for the age over seven years were extrapolations. The equation adjusted by [23] was based on information from 45 sampling plots, with time elapsed post-fire from two to 10 years, which allowed estimating biomass values up to 14 years. After this age, the model originated decreasing values, as can be seen in the curve inflection depicted in Figure 4. The new model presented in this study was adjusted to data from 150 sampling plots and allowed reasonable extrapolations between 15 and 17 years. In the specific case of forest fires in Portugal, which have fire cycles of 10 to 15 years, this new equation allowed estimating biomass accumulation after fire for that period. It is worthwhile mentioning that shrublands are systems that are not usually managed, nor particularly disturbed. As age increases, the same happens with their biomass accumulation. The large dataset used in this study allowed evidencing this trend (with the proportion of variance explained of nearly 0.90). Since all this research was derived using GIS techniques, the model could be assigned to burnt area maps and depicted as spatial biomass maps, as shown in Figure 5.

Using GIS facilities and making burnt area times post-fire accumulated biomass a function of the time elapsed after the fire, it could be verified that in the north of Portugal, the total accumulated biomass was 4,947,428 Mg in former burnt areas.

The installation of a large number of sampling plots and fieldwork for data collection and measurement are time consuming and costly, which, in large areas, may limit the studies’ feasibility. Therefore, the use of expeditious and low-cost techniques, such as the use of allometric equations to quantify shrub biomass, is a good alternative for estimating plant biomass.

Although altitude was identified as a variable that controlled shrub canopy land cover, the time elapsed after a fire was identified as the variable with a major impact on the assessment of shrub biomass.

It is worthwhile mentioning that there are no barriers related to GIS utilization in shrub biomass location and calculation tasks, as evidenced by the numerous articles written on the subject, e.g., [34,35,36,37,38,39,40]. The major constraints to the shrubs’ biomass use after GIS calculation are related to the costs of cutting, removing, and transporting biomass from the forest areas to the places of use. The amount paid for biomass does not offset the cost of exploration. Previous published results, e.g., [34], recommended a maximum distance of 35 km from biomass harvesting places to power plants. Today, due to the price of fuel, tolls, and minimum wage, the maximum distance for exploration and transportation is reduced to less than half (15 km). It is not for lack of information (satellite images and geo-referenced data) that the forest accumulates in the forests, but it is for the lack of policies to enhance biomass value as a raw material.

## 5. Conclusions

The achieved results enable stating that the woody shrub species, which are regenerated after the fires, reach high values of biomass in a few years. These results point to the remarkable ecological dynamics of this stratum and potential interest for commercial purposes.

The allometric equation presented was suitable to apply to former burnt areas within North Portugal, with post-fire ages ranging from one to 17 years, to predict the amount of accumulated shrub biomass using non-destructive methods, given the time elapsed after the fire.

When used with remote sensing or GIS techniques, it allows creating maps of shrub biomass accumulated in former burnt areas and to analyze its spatial distribution. This issue is of particular importance because it will enable locating where there is more accumulated biomass. In this way, it is possible to know where the fire hazard is higher and to pay particular attention to these places in surveillance actions or to identify where biomass can be harvested for energy purposes. In both cases, the procedures will protect the forested land. It can be highlighted that the methodology presented in this study can be straightforwardly replicated in other regions.

## Figures and Tables

**Table 1:** Allometric equations for shrub biomass estimation presented by different authors ($b_{s}$ —shrub biomass in kg shrub^−1^; $B_{s}$—generic shrub biomass in Mg ha^−1^; $B_{sc}$—shrub canopy biomass in g m^−2^; $h_{s}$—total height of the shrubs (cm); $h_{c}$—tree canopy height (m); t—time after last fire; CD—shrub canopy closure (%); h—total height of the tree (m)); and sp. is the abbreviation used for singular species.

Equation Number	Allometric Model (*Shrub Species*)	Reference
(1) (2) (3) (4) (5) (6)	$B_{sc}=9.43\;t^{2}$ − 184.11 *t* + 1034.2 (*Erica* sp.) $B_{sc}=27.54\;t^{1.202}$ (*Cistus ladanifer* L. and *Erica* sp.) $B_{sc}=0.064\;t^{2}+79.39\;t\;-76.42$ (*Cistus ladanifer* L.) $B_{sc}=1.58\;t^{2.0714}$ (*Genista and Ulex*) $B_{sc}=28.06\;t^{1.3868}$ (*Mancha*) $B_{sc}=1.58\;e^{0.088\;t}$ (*Pistacia lentiscus* L.)	[25]
(7) (8) (9)	$b_{s}=0.1239\;h^{1.1091}$ $b_{s}=6.2667{\left(\ln t\right)}^{2.040}$ $b_{s}=0.0258\;{\left(CDh_{s}\right)}^{0.754}$	[22]
(10) (11) (12)	$B_{s}=2.002-1.211\;t+0.553\;t^{2}-0.0241\;t^{3}$ $B_{s}=\exp\;\left(-1.298+1.861\;\ln t+0.1265\;\ln h\right)$ $B_{s}=\exp\;\left(2.070+0.504\;\ln t+0.057\;\ln h_{s}+\ln B_{sc}\right)$	[23]
(13) (14) (15)	$b_{s}=5.6680+0.00008\;{\left(h_{s}CD\right)}^{2}$ $b_{S}=37.634+1.010\;t$ $b_{s}=5.005+0.136\;t$	[24]

**Table 2:** Average (mean ± standard deviation (sd)) and range values of biomass accumulation (Mg ha^−1^) within 15 years after the fire and the respective percentage of canopy cover in the same time interval.

Past Time after the Fire (Years)	30% Moisture Shrub Biomass (Mg ha^−1^)		Canopy Cover (%)	
	Mean ± sd	Min − Max	Mean ± sd	Min − Max
1	0.119 ± 0.027	0.075 − 0.152	0.05 ± 0.04	0.01 − 0.10
2	0.773 ± 0.357	0.266 − 1.57	0.19 ± 0.04	0.12 − 0.23
3	2.595 ± 0.704	1.519 − 3.569	0.34 ± 0.07	0.24 − 0.44
4	4.247 ± 0.886	2.545 − 5.945	0.38 ± 0.02	0.35 − 0.41
5	6.743 ± 1.573	3.451 − 9.48	0.44 ± 0.01	0.42 − 0.45
6	8.556 ± 2.966	3.175 − 12.033	0.48 ± 0.02	0.46 − 0.50
7	9.586 ± 2.397	5.829 − 13.943	0.56 ± 0.02	0.54 − 0.59
8	13.783 ± 3.243	8.124 − 19.126	0.70 ± 0.06	0.64 − 0.77
9	15.368 ± 4.67	5.464 − 21.407	0.81 ± 0.04	0.77 − 0.87
10	19.368 ± 3.282	12.889 − 25.341	0.95 ± 0.03	0.92 − 1.00
11	21.183 ± 3.288	16.271 − 28.656	0.90 ± 0.1	0.80 − 1.00
12	25.285 ± 4.108	14.645 − 30.818	0.98 ± 0.04	0.90 − 1.00
13	25.544 ± 4.089	18.091 − 32.129	0.69 ± 0.17	0.60 − 100
14	26.871 ± 4.763	19.089 − 34.475	0.66 ± 0.23	0.50 − 1.00
15	28,877 ± 4.427	22.265 − 37596	0.78 ± 0.23	0.50 − 1.00
